# Distinct limbic connectivity in left and right benign mesial temporal lobe epilepsy: Evidence from a resting state functional MRI study

**DOI:** 10.3389/fneur.2022.943660

**Published:** 2022-09-29

**Authors:** Chiara Pizzanelli, Ilaria Pesaresi, Chiara Milano, Paolo Cecchi, Lorenzo Fontanelli, Sara Giannoni, Filippo Sean Giorgi, Mirco Cosottini, Enrica Bonanni

**Affiliations:** ^1^Department of Clinical and Experimental Medicine, Neurology Unit, University of Pisa, Pisa, Italy; ^2^Neuroradiology Unit, Pisa University Hospital, Pisa, Italy; ^3^Department of Translational Research and New Technologies in Medicine and Surgery, Neuroradiology Unit, University of Pisa, Pisa, Italy; ^4^Department of Translational Research and New Technologies in Medicine and Surgery, Human Anatomy, University of Pisa, Pisa, Italy

**Keywords:** limbic connectivity, resting state—fMRI, mesial temporal epilepsy, neuropsychological data, drug sensitive

## Abstract

**Background:**

Functional connectivity (FC) studies showed that pharmaco-resistant mesial temporal lobe epilepsy (MTLE) affects not only the limbic system, but also several extra-limbic regions, including areas belonging to resting state networks. Less is known about FC in subjects with benign MTLE (i.e., sensitive to antiseizure medication, bMTLE).

**Aim and methods:**

We evaluated FC of hippocampus and amygdala in subjects with bMTLE, distinguished based on the epileptic focus lateralization. We enrolled 19 patients (10 with left and 9 with right bMTLE) and 10 age-matched healthy subjects. Connectivity was investigated at rest by using a seed-based regression analyses approach with four regions of interest (left and right hippocampus, left and right amygdala). Patients were also tested with a neuropsychological battery and their scores were correlated with fMRI data.

**Results and conclusions:**

Our study documented an asymmetrical disruption of FC in bMTLE, in relation to the side of the focus. Right subjects only exhibited limited altered connections, while left subjects—who performed worse in verbal memory tests—showed a wide bilateral hypoconnectivity of hippocampus and amygdala with areas belonging to language and memory network. The strength of FC between left limbic areas and language and memory network correlated with better performances in verbal memory tests. Moreover, we observed an increased FC with areas of default mode network, more pronounced in left subjects, a possible attempt to compensate cognitive deficit but without effectiveness.

We believe that these findings could help to better characterize bMTLE, in which a dysfunction of limbic connectivity is detectable despite well-controlled epilepsy.

## Introduction

Mesial temporal lobe epilepsy (MTLE) is the most common type of focal epilepsy in adulthood (1, 2). Approximately one third of patients with MTLE are drug-resistant, i.e., continue to experience seizures despite at least two appropriate therapeutic attempts (3), while two thirds of subjects suffer from a milder form, which is sensitive to antiseizure medications.

Since the drug-resistant MTLE has been extensively studied over the last two decades, a large amount of neuropsychological and neuroradiological data have been provided, contributing in the characterization of the corresponding epileptic syndrome. Cognitive deficits have been well documented in severe MTLE (4) and possibly related to a disrupted connectivity between the epileptic hippocampus and several extratemporal areas involved in the control of higher order brain functions (5–7). Indeed, resting state functional Magnetic Resonance Imaging (rs-fMRI) studies performed in subjects with severe MTLE revealed disrupted connectivity involving particularly the default mode network (DMN), which is fundamental in cognitive processes.

For instance, by using independent component analysis (ICA), Zhang et al. observed decreased functional connectivity (FC) at rest in the dorsal mesial prefrontal cortex, mesial temporal lobe and inferior temporal cortex of MTLE subjects (4). Similar results were obtained by Voets et al. with the same ICA approach, documenting a reduced FC between the hippocampus, anterior temporal, precentral cortices and DMN in refractory MTLE (5). Moreover, the involvement of areas belonging to DMN was also found by Pittau et al. using a region of interest (ROI) approach and placing the ROI in hippocampus and amygdala; the Authors found that limbic regions of patients with severe MTLE were less connected with posterior cingulate cortex, precuneus and other areas of DMN (6).

Despite the abundant fMRI literature in drug-resistant MTLE, to date only 3 studies included drug-sensitive MTLE (i.e., benign MTLE, bMTLE) patients, with conflicting results possibly due to reduced sample size, heterogeneous selection criteria and different methodological approaches.

In detail, analyzing FC between nodes of DMN in 12 drug-sensitive and 15 drug-resistant patients—with both mesial and lateral temporal lobe epilepsy (TLE) - Ofer et al. found a reduced local efficiency within the DMN of drug-sensitive with respect to drug-resistant subjects, irrespectively of focus lateralization (7). However, by using a restricted ROI-based approach to compare 7 left drug-sensitive and 8 left drug-resistant TLE patients, Pressl et al. did not observe altered FC between ROIs of DMN, but rather an altered thalamo-hippocampal FC in drug-resistant compared to drug-sensitive subjects (8). Finally, the more recent study by Lee et al., using graph theory analysis on a larger number of subjects with hippocampal sclerosis, obtained results which were conflicting with the other two studies (9). In fact, only the intrinsic hippocampal connectivity, i.e., the connectivity between subregions of hippocampus, was different in drug-resistant and drug-sensitive subjects, while other measures evaluating global brain network with nodes also in the DMN and thalamic network were comparable in the two groups. In addition, the Authors did not consider the focus lateralization.

When directly comparing drug-sensitive patients and healthy controls (HC), Pressl et al. and Lee et al. did not find differences in FC parameters (8, 9), while Ofer et al. found that interconnectivity within the DMN was altered in seizure-free patients with respect to HC (7).

Of note, two of these studies were not specifically focused on MTLE but included patients with mesial and lateral TLE together. Furthermore, none of these 3 studies explored the association between FC findings and neuropsychological profile.

Given this background, and differently from previous studies which were structured to compare drug-sensitive and drug-resistant patients, the aim of this study was to investigate the resting state FC specifically in subjects with bMTLE (grouped on the basis of the epileptic focus lateralization) with respect to age-matched HC, and to correlate FC data with neuropsychological scores. These analyses might contribute to better define bMTLE as a specific clinical entity within the spectrum of MTLE.

## Methods

### Participants

Nineteen bMTLE patients (14 females and 5 males; 39.9 ± 13.6 years) were consecutively recruited at the Center for Diagnosis and Treatment of Epilepsy of the Neurology Unit of the Azienda Ospedaliero-Universitaria Pisana. All patients fulfilled the following criteria: (i) ictal semiology suggestive of MTLE (i.e., autonomic symptoms, cognitive or emotional symptoms, olfactory hallucinations at the beginning of the seizures, followed by impaired awareness, staring and automatisms); (ii) ictal or interictal EEG consistent with the diagnosis of MTLE (i.e., ictal EEG showing epileptic activity beginning in temporal regions or interictal EEG showing focal spikes or spike-and-wave activity in temporal or fronto-temporal regions); (iii) unilateral epileptogenic temporal focus; (iv) seizure freedom of at least 24 months [i.e., bMTLE according to the definition of Labate et al. (10)]; (v) right-handed according to Edinburgh Handedness Inventory.

Exclusion criteria were: (i) any suggestion of seizure onset outside the mesial temporal structures; (ii) evidence of bilateral EEG abnormalities or bilateral hippocampal atrophy; (iii) psychiatric comorbidities.

Among the 19 patients with bMTLE, 10 subjects had a left epileptic focus while 9 had a right epileptic focus.

Ten HC—matched for age, sex, handedness, and educational attainment—were enrolled.

The study was approved by the local Ethics Committee in accordance with the Helsinki Declaration. Each subject gave a written informed consent to participate in the study.

### Clinical features and neuropsychological evaluation

Demographic and clinical features recorded for each subject included sex, age, educational attainment, age at seizure onset, duration of the disease, number of antiseizure medications (ASMs) taken at the time of the enrollment, and evidence of hippocampal sclerosis (HS) on MRI.

Within 3 months from recruitment, all participants underwent a battery of standardized neuropsychological tests, which included: Rey Auditory Verbal Learning Task (RAVLT) with immediate and delayed recall, to assess verbal learning and verbal memory (11); Short Tale Test (STT), with immediate and delayed recall, to evaluate episodic memory (12); Rey-Osterrieth Complex Figure test (ROCF) (12, 13), with immediate and delayed recall, to investigate visuospatial functions and memory; Trail making test (TMT) (14) and Stroop Interference Test (15) to explore selective attention and inhibition.

Demographic, clinical, and neuropsychological features of the two groups with left and right bMTLE were compared using *t*-test for continuous variables and Fisher's exact test for categorical variables.

### Data acquisition

Within 3 months from enrollment, all subjects underwent an MRI scan at the Neuroradiology Unit of the University of Pisa.

Images were acquired on a 3T scanner (Discovery MR750 3T, GE Healthcare, Milwaukee) with high-performing gradients (strength 50 mT/m, maximum slew rate 200 T/m/s) equipped with an eight channels head coil with ASSET technology. Rs-fMRI data were acquired using a T2^*^ weighted gradient recalled echo-planar imaging (EPI) sequence (TR 2500 ms, TE 40 ms, flip angle 90°, FOV 260 mm, matrix size 128 x 128) with 28 interleaved slices (thickness 4 mm, spacing 1 mm) angled of 30° with respect to the anterior-posterior commissural plane (AC-PC) to minimize susceptibility artifacts, repeated over 200 volumes (total scanning time of 8'20”). A high-resolution 3D Spoiled Gradient Recalled sequence was also acquired on sagittal plane (TR 8.2 ms; TE 3.2 ms; flip angle 12°; TI 450 ms; FOV 256 mm; matrix size 256 x 256; 178 slices; thickness 1 mm). Foam cushions were used for head stabilization to reduce motion-related artifacts. We asked subjects to maintain their eyes closed and to remain awake.

### Voxel-based morphometry

In order to take into account possible regional atrophy in bMTLE patients, we performed an automated analysis of T1 structural data by an optimized VBM protocol (16) carried out with FMRIB software library package (FSL) (17). Structural images were brain-extracted using BET (Brain Extraction Tool) (18), and then they were automatically segmented into gray matter (GM), white matter (WM), and cerebrospinal fluid (CSF) by FMRIB's Automated Segmentation Tool (FAST) (19). The GM volume images were aligned to the Montreal Neurological Institute (MNI) 152 standard space (20) by the affine registration (FLIRT tool) (21), followed by non-linear registration using FNIRT (22). The registered GM images were averaged and flipped along the x axis to create two left-right symmetric, study-specific gray matter templates: Template 1, obtained by HC and left bMTLE patients; Template 2, obtained by HC and right bMTLE patients.

All native GM images were non-linearly registered to the appropriate study-specific template (i.e., Template 1 for left bMTLE vs. HC comparison, Template 2 for right bMTLE vs. HC comparison), modulated and smoothed with an isotropic Gaussian kernel with a sigma of 3 mm. Statistical analyses were performed using different voxel-wise General Linear Model (GLM) with a permutation-based non-parametric testing (5,000 permutations), corrected for multiple comparisons across space (*p* < 0.05) by threshold-free cluster enhancement (TFCE) option (23). Age and gender of patients and HC were inserted as covariate variables within each GLM matrix (24).

### Resting state fMRI (rs-fMRI) data pre-processing

Resting state functional data were analyzed by FEAT tool part of FSL. The first four scans of each fMRI run were discarded from analysis to avoid saturation effect of magnetization. Image preprocessing also included removal of non-brain structures (by using BET) (18), slice-timing correction, motion correction (by using MCFLIRT) (21), spatial smoothing (Gaussian kernel of Full Width Half Maximum = 8 mm) and high pass temporal filtering (cut off = 100.0 s). Subjects with absolute translational or rotational displacement higher than 3 mm or 3 degrees were excluded from further analysis.

In order to remove artifactual signal components from subsequent analyses, we extracted the mean timecourses for WM and CSF masks for each subject. In more detail, WM and CSF images, obtained with FAST segmentation, were thresholded at 80% tissue type probability to create WM and CSF masks. These masks were registered to functional images by affine transformation, then they were applied to the preprocessed functional image in order to extract the mean timecourses for WM and CSF averaging the time series of all voxels within the masks. The single-subject time series were then used as nuisance regressors in the statistical model.

### Seed-based functional connectivity analysis

Functional connectivity analysis was performed by a seed-based regression approach. Seed regions were obtained by an automated segmentation tool, FIRST (part of FSL), which extracted four ROIs from T1 structural data of each subject: right hippocampus, left hippocampus, right amygdala and left amygdala. These ROIs were registered to single-subject functional images space by affine transformation with FLIRT (21), then they were applied to the preprocessed functional images in order to extract the mean timecourses of each seed.

For each subject, first-level statistical analysis was carried out by four multiple regression analyses (using General Linear Model implemented in FEAT) with a seed timecourse regressor (left hippocampus, right hippocampus, left amygdala, right amygdala, separately) and 8 nuisance covariates (6 motion parameters, CSF signal and WM signal). Each statistical correlation map was registered by affine transformation to MNI template and both positive and negative BOLD changes were considered for the second-level analyses.

Between-group analyses were performed by a Mixed Effect model approach (25, 26). The following comparisons were carried out: left bMTLE vs HC, right bMTLE vs HC. Corrections for multiple comparisons were applied at the cluster level by using Gaussian random field theory (min *Z* > 2.3; cluster significance: *p* < 0.05). The results were adjusted for age and sex.

### Relationships between FC strength and clinical/neuropsychological features

Pooling all bMTLE patients together regardless of side of the epileptic focus, we investigated the relationships between FC strength, clinical features and neuropsychological tests. We implemented one sample *t*-test by Mixed Model in FSL by inserting clinical data or test scores as covariate variables within different GLM design matrix. The resulted Z statistical maps were thresholded using clusters determinated by *Z* > 2.3 and a corrected cluster significance threshold of *p* < 0.05.

## Results

### Clinical features and neuropsychological evaluation

Demographic and clinical features of left and right bMTLE patients are illustrated in Table 1.

**Table 1:** Demographic, clinical, and neuropsychological features.

	**Right bMTLE (*n =* 9)**	**Left bMTLE (*n =* 10)**	**HC (*n =* 10)^§^**	***P*-value Right vs. left bMTLE**
**Demographic and clinical features**
Age (years)	37.56 ± 14.81	42.10 ± 12.90	39.70 ± 11.50	0.484
Sex (F/M)	7/2	7/3	6/4	0.990
Schooling (years)	14.44 ± 1.81	13.80 ± 2.90	14.20 ± 2.30	0.574
Age at onset (years)	26.11 ± 11.45	29.70 ± 14.48		0.560
Disease length (years)	11.44 ± 5.22	12.40 ± 13.11		0.840
Number of ASMs	1.11 ± 0.33	1.20 ± 0.42		0.615
Presence of HS	5/9	4/10		0.459
**Neuropsychological scores**
RAVLT immediate recall	43.59 ± 6.12	35.97 ± 10.43		0.086
RAVLT delayed recall	9.41 ± 1.95	6.47 ± 2.61		0.024^*^
STT immediate recall	6.83 ± 1.02	5.26 ± 1.64		0.045^*^
STT delayed recall	5.94 ± 1.43	4.07 ± 1.78		0.043^*^
ROCF immediate recall	15.31 ± 5.48	15.24 ± 5.39		0.980
ROCF delayed recall	15.50 ± 4.92	17.03 ± 6.14		0.588
Stroop Test IET	22.37 ± 7.82	19.97 ± 6.99		0.516
Stroop Test IEE	0.53 ± 1.00	0.69 ± 1.39		0.784
TMT A	45.87 ± 12.55	46.22 ± 11.88		0.954
TMT B	113.75 ± 27.79	92.67 ± 24.55		0.121
TMT B-A	69.62 ± 22.69	45.33 ± 18.32		0.090

Results were expressed using mean ± standard deviation for continuous variables and relative frequency for categorical variables. Demographic, clinical, and neuropsychological features were compared using t-test for continuous variables or Fisher's exact test for categorical variables, as appropriate.

bMTLE, benign mesial temporal lobe epilepsy; ASMs, antiseizure medications; HC, healthy controls; HS, hippocampal sclerosis; RAVLT, Rey Auditory Verbal Learning Task; STT, Short Tale Test; ROCF, Rey-Osterrieth Complex Figure test; IET, interference effect time; IEE, interference effect errors; TMT, Trail Making Test.

§ HC were matched for age, sex, handedness, and educational attainment.

* Denotes significant difference between groups (p < 0.05).

There were no significant differences between the two groups in terms of age, sex, educational attainment, age at seizure onset, duration of the disease, number of ASMs, and evidence of HS.

The results of the neuropsychological evaluation are also summarized in Table 1. Patients' performance was in the normal range; however left bMTLE patients showed worse performance compared to right bMTLE patients in RAVLT delayed recall and STT immediate and delayed recall.

### Voxel-based morphometry

The between-group VBM analyses revealed no significant differences in cortical GM volume both between left bMTLE patients and HC, and between right bMTLE and HC.

### Seed-based FC analysis: Left bMTLE

**Figure 1:**
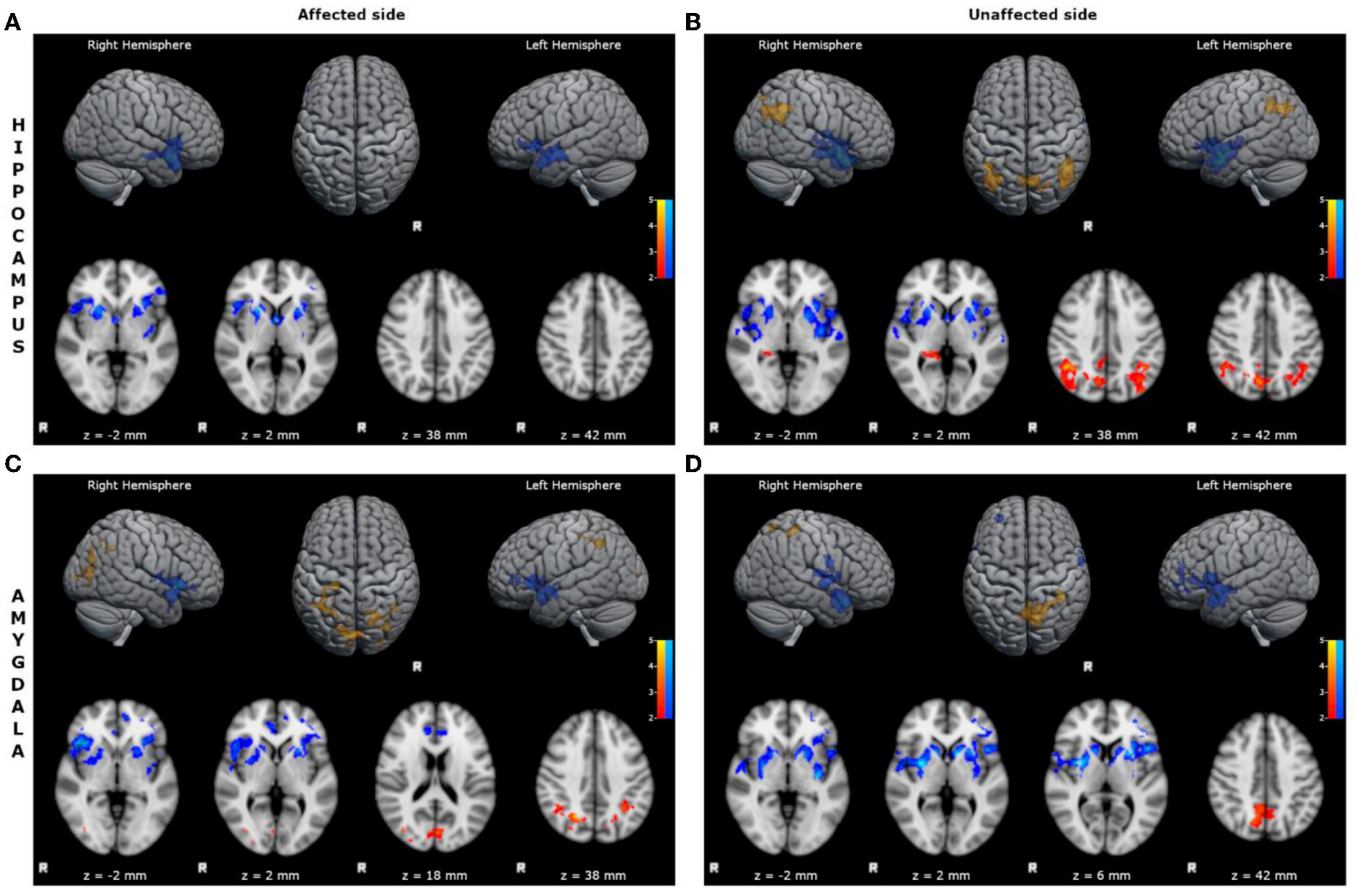
Statistical comparison maps between left bMTLE patients and HC for seeds in left hippocampus **(A)**, right hippocampus **(B)**, left amygdala **(C)**, right amygdala **(D)**. Reduced FC is shown in blue, while increased FC is shown in orange and red. Results are superimposed on axial slices and volumes of standard MNI template (Z threshold > 2.3, cluster p significance < 0.05). **(A)** FC analysis showed reduced connectivity of left hippocampus with bilateral inferior frontal cortices, limbic structures, superior and mid temporal cortices and basal ganglia. **(B)** FC analysis showed reduced connectivity of right hippocampus with bilateral inferior frontal cortices, limbic structures, superior and mid temporal cortices and basal ganglia; and increased connectivity of right hippocampus with bilateral mid-posterior cinguli, angular gyri, precunei, and parieto-occipital cortices. **(C)** FC analysis showed reduced connectivity of left amygdala with bilateral superior and inferior frontal cortices, limbic structures, superior and mid temporal cortices, and basal ganglia; and increased connectivity of left amygdala with bilateral precunei and parieto-occipital cortices. **(D)** FC analysis showed reduced connectivity of right amygdala with bilateral superior and inferior frontal cortices, limbic structures, superior and mid temporal cortices, and basal ganglia; and increased connectivity of right amygdala with bilateral precunei, right cingulum and parieto-occipital cortex.

Benign MTLE patients with left epileptic focus showed with respect to HC:

- reduced FC of the affected hippocampus with bilateral inferior frontal cortices, limbic structures, superior and mid temporal cortices, and basal ganglia (Figure 1; Supplementary Table S1);
- reduced FC of the unaffected hippocampus with bilateral inferior frontal cortices, limbic structures, superior and mid temporal cortices, and basal ganglia (Figure 1; Supplementary Table S2);
- increased FC of the unaffected hippocampus with bilateral mid-posterior cinguli, angular gyri, precunei, and parieto-occipital cortices (Figure 1; Supplementary Table S3);
- reduced FC of the affected amygdala with bilateral superior and inferior frontal cortices, limbic structures, superior and mid temporal cortices, and basal ganglia (Figure 1; Supplementary Table S4);
- increased FC of the affected amygdala with bilateral precunei and parieto-occipital cortices (Figure 1; Supplementary Table S5);
- reduced FC of the unaffected amygdala with bilateral superior and inferior frontal cortices, limbic structures, superior and mid temporal cortices, and basal ganglia (Figure 1; Supplementary Table S6);
- increased FC of the unaffected amygdala with bilateral precunei, right cingulum, and parieto-occipital cortex (Figure 1; Supplementary Table S7).

### Seed-based FC analysis: Right bMTLE

**Figure 2:**
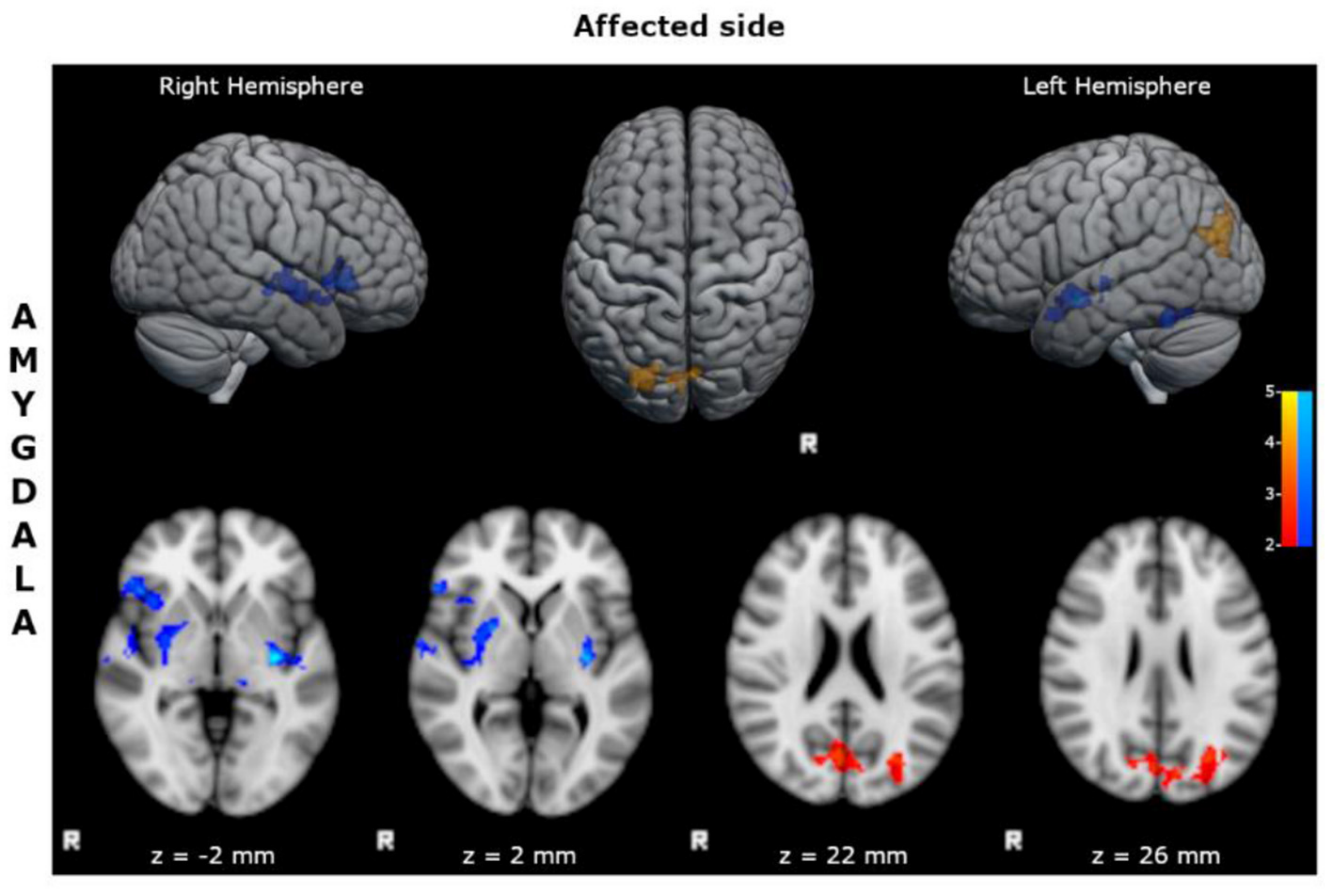
Statistical comparison maps between right bMTLE patients and HC for seed in right amygdala. Reduced FC is shown in blue, while increased FC is shown in orange and red. Results are superimposed on axial slices and volumes of standard MNI template (Z threshold > 2.3, cluster p significance < 0.05). FC analysis showed reduced connectivity of right amygdala with bilateral inferior frontal cortices, superior and mid temporal cortices, limbic structures, and basal ganglia; and increased connectivity of right amygdala with precunei and bilateral parieto-occipital cortices. No changes in FC were detected for seeds in right hippocampus, left hippocampus and left amygdala.

Benign MTLE patients with right epileptic focus showed with respect to HC:

- reduced FC of the affected amygdala with bilateral inferior frontal cortices, superior and mid temporal cortices, limbic structures, and basal ganglia (Figure 2; Supplementary Table S8);
- increased FC of the affected amygdala with precunei and bilateral parieto-occipital cortices (Figure 2; Supplementary Table S9).

### Relationships between hippocampal FC and clinical/neuropsychological features in bMTLE patients

**Figure 3:**
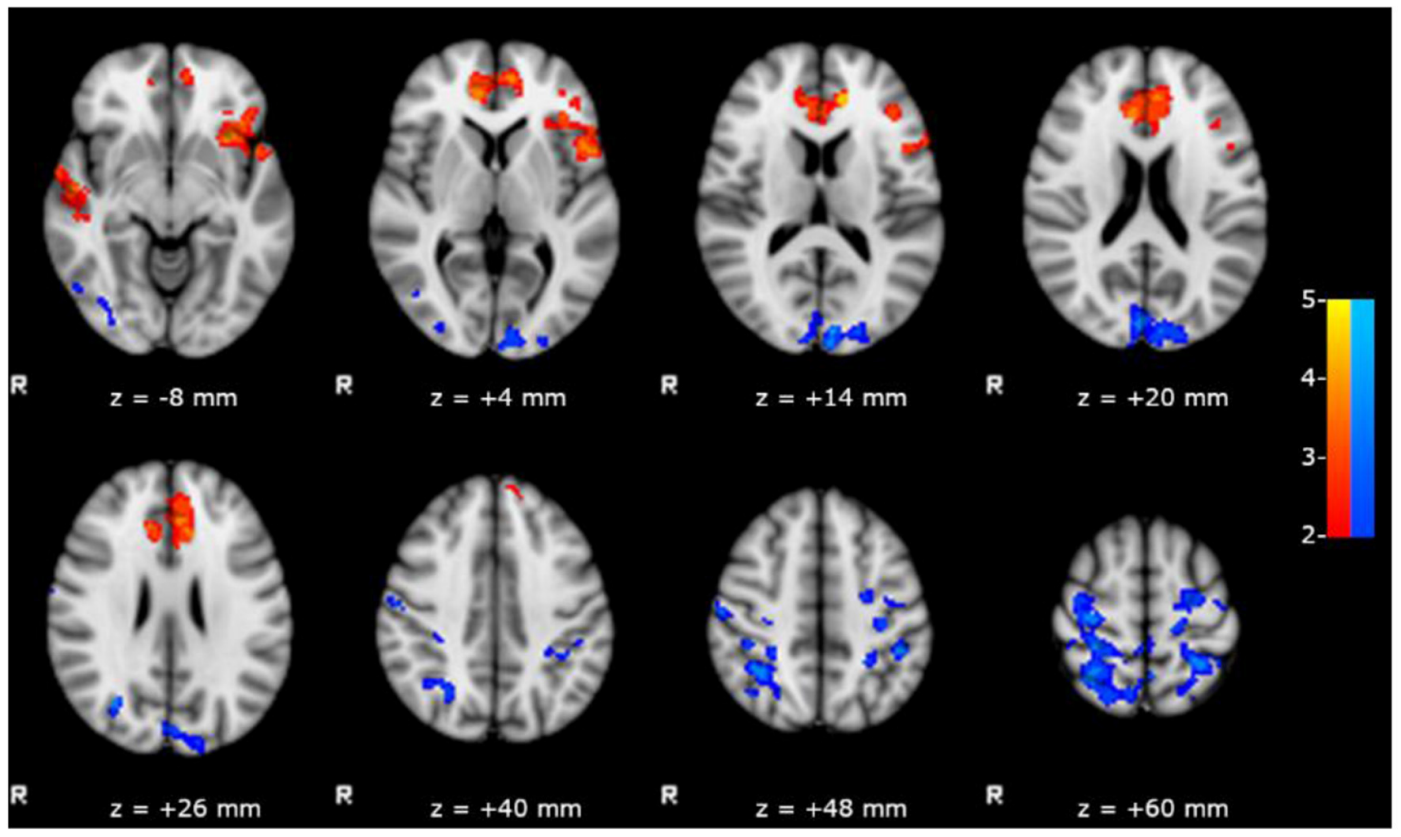
Results of the within group analysis in bMTLE patients for FC of left hippocampus obtained introducing RAVLT delayed recall test score as covariate. Results are superimposed on axial slices of standard MNI template (Z threshold > 2.3, cluster p significance < 0.05). A significant positive relationship (in red) between the RAVLT delayed recall test and the FC of left hippocampus was observed in bilateral inferior frontal and superior temporal gyri. A significant negative relationship (in blue) between the RAVLT delayed recall test and the FC of left hippocampus was observed in posterior DMN areas.

We observed a significant positive correlation between the scores obtained in RAVLT delayed recall test and the FC of left hippocampus with bilateral inferior frontal, anterior cinguli, and superior temporal gyri (Figure 3), and a negative correlation between the same scores and the FC of left hippocampus with posterior DMN.

No other significant relationships between resting state FC and clinical/neuropsychological features were found.

## Discussion

The aim of this study was to explore differences in resting-state FC in patients with bMTLE compared to HC, by using a ROI-based approach.

As a main finding, we documented a marked asymmetry of FC between left and right bMTLE: left bMTLE patients showed an extended pattern of disrupted connections between limbic structures and distant brain areas, while right bMTLE subjects only exhibited a limited alteration of FC.

This asymmetry has already been reported by several Authors in drug-resistant MTLE (27, 28). For instance, a resting state fMRI study by Pereira et al. revealed a marked impairment of FC in left MTLE and slight FC changes in right MTLE (29). Other studies obtained comparable results by adopting graph theoretical analysis, i.e., they revealed diffuse network disruptions in left but not in right MTLE (30, 31). Therefore, left MTLE seems to underlie more intricate bilateral disfunctions than right MTLE (28).

The reasons for these lateralization-related differences could rely on the intrinsic cerebral asymmetry of human brain, which is responsible for the functional organization of most cognitive systems (32). Due to its prominent role in language function, the left cerebral hemisphere undergoes a longer period of network maturation, which may thus make it more vulnerable in case of epilepsy (33). Namely, repeated epileptic seizures involving the left cerebral hemisphere might produce more pronounced disruptions of FC in comparison to the right one.

A further point to underline is that we found FC alterations in patients who were seizure-free. It is well known that recurrent seizures can modify networks connected to epileptic circuitry by neuroplasticity processes that takes place over many years (34, 35). Therefore, a previous history of repeated seizures, even if far in the past, may produce durable altered connections between limbic structures and remote regions, and subsequent prolonged seizure freedom may not be sufficient to allow reestablishment of normal FC.

Our findings are quite different from those obtained by the other three fMRI studies involving patients with bMTLE (7–9): first, we were able to detect differences in FC between bMTLE and HC, while both Pressl et al. and Lee et al. did not document any difference between drug-sensitive patients and HC, but only between drug-resistant and drug-sensitive subjects (8, 9). Instead, in the study by Ofer et al., FC of seizure-free subjects was different from that of not seizure-free and that of HC but, surprisingly, parameters of FC were similar in drug-resistant patients and HC (7).

Nevertheless, it is very arduous to compare those results to ours, due to the different methodology used to analyze FC, which were ROI-to-ROI analyses (8), graph-theoretical analyses (7, 9), and ROI-based analyses with seed in hippocampus and amygdala for us. Even more importantly, in two out of three studies, patients with mesial and lateral TLE were enrolled together, and the lateralization of the epileptic foci was not considered, while our study is specifically focused on MTLE patients, distinguished in left and right.

Below, we will discuss more in detail the specific findings of the study.

### Decreased connectivity toward language-and-memory network

In our series, both affected and unaffected hippocampus and amygdala of patients with left bMTLE exhibited a decreased FC with bilateral inferior frontal areas, limbic structures, and temporal neocortices (Figure 1). These regions belong to language-and-memory network (LMN), a complex brain system mainly located in the dominant hemisphere, which groups together cortical areas responsible for verbal memory and for basic language skills (36–38).

A similar pattern of decreased FC was found in right bMTLE, although the alterations were less wide, and only involved the FC of the right amygdala (Figure 2).

Previous fMRI studies have investigated resting-state FC changes in regions and hubs traditionally involved in LMN. For instance, in 2006 Waites et al. first raised the question of a language network dysfunction in left MTLE, observing a reduction of FC between different language and verbal memory areas in MTLE patients compared to HC (39).

More recently, the relationship between hippocampus and LMN areas was specifically investigated by Roger et al., who documented a hypoconnectivity at rest between the affected hippocampus and fronto-temporo-parietal regions belonging to the LMN. Such hypoconnectivity was observed in both left and right bMTLE patients, but it was more pronounced in left ones (36). Similar results were obtained by Whitten et al. who demonstrated that MTLE impacts hippocampal networks relevant for language processing by potentially decreasing recruitment of the ipsilateral epileptic hippocampus (40).

Verbal memory and language impairment is one of the most common cognitive complaints in patients with MTLE. In particular, these verbal difficulties—characterized by struggles in memorizing words, naming and fluency—have been extensively studied in drug-resistant MTLE patients (41–43) and appear to be more pronounced in subjects with left MTLE (44, 45). Albeit to a lesser extent, more recent neuropsychological studies documented a slight verbal memory and language impairment also in bMTLE (46, 47).

Although there are no fMRI studies on bMTLE specifically exploring LMN, based on our findings, we could assume that the disrupted connections toward LMN may represent the functional substrate responsible for the slight language impairment described in bMTLE.

In line with this hypothesis, our left bMTLE patients—who showed a more pronounced reduction of FC toward LMN areas compared to right bMTLE—obtained significant lower scores in neuropsychological tests exploring verbal memory. Moreover, the strength of FC between left hippocampus and LMN positively correlated with performances in RAVLT delayed recall, a test exploring verbal learning and memory, thus confirming the importance of these connections in verbal tasks.

Of interest, in our study also the unaffected hippocampus and amygdala in left bMTLE exhibited a pattern of disrupted connections toward LMN areas.

This is in line with a previous study by Pittau et al., in which the Authors described the same altered FC for seeds in both affected and unaffected hippocampus/amygdala (6).

Also Pereira et al. found altered connections of the unaffected hippocampus, and demonstrated that this pattern of disrupted connectivity diverged between left and right MTLE patients, being more pronounced in left MTLE group (29).

In accordance with literature, our results might suggest that functional brain plasticity (also involving the contralateral side) might have occurred in bMTLE patients, with significant differences related to the epileptic focus lateralization.

### Decreased connectivity toward basal ganglia network

Another intriguing result of our study is the altered FC of hippocampus and amygdala toward subcortical structures (Figure 1). In detail, affected and unaffected hippocampus and amygdala in left bMTLE, and affected amygdala in right bMTLE showed decreased FC with basal ganglia network (BGN). This network refers to a complex group of interconnected subcortical nuclei involved in motor control, cognition and motivational behaviors (48), which physiologically receives inputs from neocortex but also from hippocampus and amygdala (49). Although the BGN is not directly implicated in the initiation of seizures, it seems to contribute to the regulation and propagation of epileptic activity (49, 50), thus representing an important node of the epileptogenic network in MTLE. For instance, some ictal manifestations occurring during seizures arising from mesial temporal lobe—such as contralateral dystonic posturing or even ipsilateral upper limb automatisms—suggest an involvement of basal ganglia in seizure's propagation, that has been confirmed by ictal SPECT studies (51, 52). At the same time, several studies analyzing metabolic alterations and advanced imaging, have suggested an inhibitory role of the BGN on ictal discharge's propagation *via* its feedback pathways to the cortex (49, 53, 54). In this respect, the ictal activation of basal ganglia could represent an attempt of the brain to block seizure's progression and secondary generalization.

Concerning the significance of FC between BGN and cortical areas during interictal resting state, the available data suggest that such altered connections might reflect an impaired inhibitory function of the BGN (30, 55).

Although our patients are all drug-sensitive, we speculate that this disrupted FC between limbic structures and basal ganglia could represent a sign of the alteration in BGN due to presence of the epileptogenic network, irrespectively of the response to ASMs.

### Increased connectivity toward default mode network

In our study unaffected hippocampus and amygdala in left bMTLE and right amygdala in right bMTLE showed increased FC with areas of the posterior DMN, i.e., cinguli, precunei and parietal cortices.

Patterns of hyperconnectivity toward DMN have already been described in right MTLE patients by Zhang et al. (4) and, more recently, by Zhao et al. (56), who attributed this finding to a possible compensatory mechanism responsible for better neuropsychological performance.

Another possible explanation of increased FC is the development of new excitatory synapses and axonal sprouting, a phenomenon well documented by histological studies (57) as a consequence of the frequent generation and spreading of epileptic seizures (58). Some authors underlie that the majority of these newly formed synapsis is however aberrant rather than serving as a compensatory mechanism (59).

We highlight that in our study left bMTLE patients showed increased connections of hippocampus/amygdala with DMN areas, and that such hyperconnectivity was even more widespread than in right bMTLE patients, despite worse neuropsychological performance.

Moreover, pooling together patients with epileptogenic zones in either hemisphere, the strength of FC between left hippocampus and DMN negatively correlated with RAVLT delayed recall scores; in other words, patients with worse performance had a stronger FC between left hippocampus and DMN (Figure 3).

Thus, we could speculate that the observed resting state hyperconnectivity may more likely represent the expression of an inefficient plasticity, rather than an effective compensatory phenomenon.

### Strength and limitations of the study

To our knowledge, this is the first resting-state fMRI study conducted on bMTLE patients, which included cognitive evaluations and correlated the results of neuropsychological tests with fMRI data. However, due to the relatively small sample size, further studies are needed to confirm our findings.

Furthermore, we specify that we separately investigated the correlation differences between HC and MTLE patients at the four ROIs. Thus, due to the intrinsically high correlation among hippocampi and amygdalae of both sides, the differences observed at each ROI may be not independent from one another, but may partially share the same origin. Therefore, the obtained results should be interpreted with caution. Lastly, another limitation of this study is the lack of neuropsychological data in HC.

## Conclusions

In summary, our study documented an asymmetrical disruption of FC in bMTLE, in relation to the side of the epileptic focus. While subjects with right focus only exhibited limited decreased connections of affected amygdala toward some areas of LMN, subjects with left focus—who performed worse in verbal memory tests—showed a wide bilateral hypoconnectivity of hippocampus and amygdala toward several areas of LMN. The strength of FC between left limbic areas and LMN correlated with better performance in verbal memory tests, thus confirming the importance of these connections in verbal memory tasks.

We believe that these findings could help in understanding bMTLE pathophysiology. In particular, our results suggest that left bMTLE patients might be more prone to the disruption of limbic connectivity induced by previous seizures and, at the same time, less able to organize compensatory strategies in comparison to subjects with right focus, with consequent worse neuropsychological functioning.

## Data availability statement

The original contributions presented in the study are included in the article/Supplementary material, further inquiries can be directed to the corresponding author.

## Ethics statement

The studies involving human participants were reviewed and approved by the Institutional Review Board of Neurology Clinic, Santa Chiara University Hospital of Pisa. The patients/participants provided their written informed consent to participate in this study.

## Author contributions

CP and IP conceived and designed the analysis. IP, PC, and SG collected and analyzed the data. CM and CP wrote the paper. CM and LF contributed to analysis tools. FG, MC, and EB made a significant contribution in reviewing the paper. All authors contributed to the article and approved the submitted version.

## Conflict of interest

The authors declare that the research was conducted in the absence of any commercial or financial relationships that could be construed as a potential conflict of interest.

## Publisher's note

All claims expressed in this article are solely those of the authors and do not necessarily represent those of their affiliated organizations, or those of the publisher, the editors and the reviewers. Any product that may be evaluated in this article, or claim that may be made by its manufacturer, is not guaranteed or endorsed by the publisher.
