# Supplementary material for: Distinct limbic connectivity in left and right benign mesial temporal lobe epilepsy: Evidence from a resting state functional MRI study
Source: Front Neurol. 2022 Sep 29;13:943660. doi: 10.3389/fneur.2022.943660 (PMC9558280; doi:10.3389/fneur.2022.943660)
Supplement: Supplementary file 1 [file Data_Sheet_1.pdf]

Table S1: Decreased left hippocampal connectivity in left bMTLE compared to HC

AAL anatomical area		Activated (mm^3)	Z max	MNI Coordinates		
				X	Y	Z
**Frontal lobe**						
12	Frontal_Inf_Oper_R	296	3.0	46	18	0
13	Frontal_Inf_Tri_L	568	3.2	-38	32	-2
14	Frontal_Inf_Tri_R	248	3.1	46	20	0
15	Frontal_Inf_Orb_L	1080	3.8	-22	8	-20
16	Frontal_Inf_Orb_R	848	3.9	44	18	-16
21	Olfactory_L	392	3.7	-22	8	-18
22	Olfactory_R	352	3.2	8	10	-18
**Limbic system**						
29	Insula_L	2632	4.0	-38	8	-14
30	Insula_R	2296	4.2	46	12	-12
41	Amygdala_L	672	3.7	-22	0	-14
42	Amygdala_R	168	3.0	24	-2	-14
39	Parahippocampal_L	320	3.2	-22	6	-24
40	Parahippocampal_R	392	4.0	16	4	-22
**Basal ganglia**						
71	Caudate_L	248	3.0	-16	12	8
72	Caudate_R	664	3.5	18	16	2
73	Putamen_L	2304	3.7	-26	14	4
74	Putamen_R	2792	4.2	28	20	0
**Temporal lobe**						
81	Temporal_Sup_L	1072	3.5	-46	-2	-10
82	Temporal _Sup_R	376	3.3	50	-4	-14
83	Temporal_Pole_Sup_L	2240	4.0	-38	12	-24
84	Temporal_Pole_Sup_R	3152	4.7	48	12	-20
85	Temporal_Mid_L	1128	3.3	-52	-10	-16
86	Temporal_Mid_R	360	3.4	48	-10	-16
87	Temporal_Pole_Mid_L	240	2.9	-48	8	-26
88	Temporal_Pole_Mid_R	96	2.8	48	10	-24

Table S2: Decreased right hippocampal connectivity in left bMTLE compared to HC

AAL anatomical area		Activated (mm^3)	Z max	MNI Coordinates		
				X	Y	Z
**Frontal lobe**						
5	Frontal_Sup_Orb_L	152	2.9	-24	10	-16
9	Frontal_Mid_Orb_L	64	2.9	-20	28	-22
11	Frontal_Inf_Oper_L	176	3.1	-46	12	0
12	Frontal_Inf_Oper_R	128	2.8	48	10	0
13	Frontal_Inf_Tri_L	56	2.7	-38	32	-2
15	Frontal_Inf_Orb_L	1240	3.9	-22	8	-20
16	Frontal_Inf_Orb_R	1144	3.7	40	26	-22
17	Rolandic_Oper_L	24	2.7	-48	8	0
18	Rolandic_Oper_R	1072	3.5	62	-2	8
21	Olfactory_L	352	3.8	-22	4	-18
**Limbic system**						
29	Insula_L	4432	4.8	-38	6	-14
30	Insula_R	3336	5.0	40	12	-14
41	Amygdala_L	976	3.7	-28	-4	-14
42	Amygdala_R	112	2.9	36	2	-24
**Basal ganglia**						
71	Caudate_L	288	3.0	-6	6	2
72	Caudate_R	400	3.3	18	16	4
73	Putamen_L	4080	3.7	-20	6	-2
74	Putamen_R	3240	3.7	22	14	4
75	Pallidum_L	128	3.9	-20	4	-2
76	Pallidum_R	432	3.6	18	8	0
**Temporal lobe**						
81	Temporal_Sup_L	2544	3.7	-42	2	-10
82	Temporal_Sup_R	1720	4.2	44	-8	-14
83	Temporal_Pole_Sup_L	2929	4.6	-20	6	-20
84	Temporal_Pole_Sup_R	3952	4.5	44	12	-16
85	Temporal_Mid_L	1720	4.4	-50	2	-24
86	Temporal_Mid_R	488	3.5	50	-16	-16
87	Temporal_Pole_Mid_L	312	3.3	-44	6	-26
88	Temporal_Pole_Mid_R	80	2.7	48	16	-26

Table S3: Increased right hippocampal connectivity in left bMTLE compared to HC

AAL anatomical area		Activated (mm^3)	Z max	MNI Coordinates		
				X	Y	Z
**Limbic system**						
33	Cingulum_Mid_L	136	2.8	0	-40	34
34	Cingulum_Mid_R	576	2.9	4	-42	32
35	Cingulum_Post_L	624	2.8	-2	-40	22
36	Cingulum_Post_R	432	3.4	6	-42	10
**Parietal lobe**						
59	Parietal_Sup_L	72	2.8	-26	-62	42
60	Parietal_Sup_R	312	2.9	24	-72	50
61	Parietal_Inf_L	1784	3.4	-44	-52	38
62	Parietal_Inf_R	976	4.3	46	-50	38
64	SupraMarginal_R	176	2.8	46	-42	36
65	Angular_L	1928	3.2	-36	-66	38
66	Angular_R	4064	3.9	40	-56	32
67	Precuneus_L	784	3.6	-6	-66	48
68	Precuneus_R	2648	3.8	6	-68	44
**Occipital lobe**						
46	Cuneus_R	64	2.6	10	-72	38
48	Lingual_R	112	2.8	12	-40	2
50	Occipital_Sup_R	96	2.8	20	-62	40
51	Occipital_Mid_L	976	3.4	-42	-78	38
52	Occipital_Mid_R	704	3.0	42	-72	34

Table S4: Decreased left amygdala connectivity in left bMTLE compared to HC

AAL anatomical area		Activated (mm^3)	Z max	MNI Coordinates		
				X	Y	Z
**Frontal lobe**						
3	Frontal_Sup_L	48	2.7	-28	48	0
5	Frontal_Sup_Orb_L	24	2.5	-22	12	-16
7	Frontal_Mid_L	240	2.9	-32	46	6
12	Frontal_Inf_Oper_R	496	3.8	48	20	-2
13	Frontal_Inf_Tri_L	976	3.6	-34	28	-2
14	Frontal_Inf_Tri_R	728	3.8	44	26	-2
15	Frontal_Inf_Orb_L	496	3.5	-34	26	-4
16	Frontal_Inf_Orb_R	1424	4.0	46	18	-12
18	Rolandic_Oper_R	248	3.3	50	6	0
21	Olfactory_L	256	3.4	-20	8	-18
23	Frontal_Sup_Medial_L	336	3.4	-12	52	0
24	Frontal_Sup_Medial_R	16	2.3	12	46	0
25	Frontal_Mid_Orb_L	704	3.7	-8	52	-8
**Limbic system**						
31	Cingulum_Ant_L	2152	4.3	2	36	14
32	Cingulum_Ant_R	1832	4.2	4	36	14
33	Cingulum_Mid_L	24	2.4	-8	28	32
29	Insula_L	3176	4.0	-28	24	2
30	Insula_R	3968	4.3	44	22	-6
**Basal ganglia**						
72	Caudate_R	344	3.3	16	14	2
73	Putamen_L	2168	3.7	-24	14	4
74	Putamen_R	1600	3.5	18	12	0
75	Pallidum_L	40	2.6	-18	6	-4
76	Pallidum_R	232	2.9	18	8	-2
**Temporal lobe**						
81	Temporal_Sup_L	536	4.3	-48	4	-10
82	Temporal_Sup_R	80	2.5	54	-8	2
83	Temporal_Pole_Sup_L	640	2.4	-48	8	-8
84	Temporal_Pole_Sup_R	720	2.7	46	14	-18
85	Temporal_Mid_L	48	2.8	-44	-4	-18
86	Temporal_Pole_Mid_R	24	2.4	34	10	-34

Table S5: Increased left amygdala connectivity in left bMTLE compared to HC

AAL anatomical area		Activated (mm^3)	Z max	MNI Coordinates		
				X	Y	Z
**Parietal lobe**						
57	Postcentral_L	392	3.0	-22	-30	56
59	Parietal_Sup_L	392	3.2	-22	-62	44
60	Parietal_Sup_R	64	2.8	18	-58	50
61	Parietal_Inf_L	2120	3.7	-42	-50	42
62	Parietal_Inf_R	192	3.1	42	-52	38
65	Angular_L	56	2.8	-32	-50	36
66	Angular_R	448	2.9	38	-58	36
67	Precuneus_L	168	3.4	-12	-72	36
68	Precuneus_R	688	4.0	20	-64	40
**Occipital lobe**						
43	Calcarine_L	1120	3.8	0	-78	16
44	Calcarine_R	344	2.7	14	-88	14
45	Cuneus_L	3032	3.7	0	-80	16
46	Cuneus_R	760	3.9	20	-64	38
47	Lingual_L	80	2.6	2	-80	4
48	Lingual_R	32	2.5	4	-78	2
49	Occipital_ Sup_L	480	2.9	-10	-94	8
50	Occipital_ Sup_R	792	4.1	24	-62	36
51	Occipital_ Mid_L	312	3.3	-26	-62	32
52	Occipital_ Mid_R	1840	4.0	34	-76	28

Table S6: Decreased right amygdala connectivity in left bMTLE patients compared to HC

AAL anatomical area		Activated (mm^3)	Z max	MNI Coordinates		
				X	Y	Z
**Frontal lobe**						
3	Frontal_Sup_L	56	2.7	-28	46	0
5	Frontal_Sup_Orb_L	336	3.1	-14	10	-22
6	Frontal_Sup_Orb_R	48	3.3	20	14	-20
7	Frontal_Mid_L	784	3.8	-32	46	6
9	Frontal_Mid_Orb_L	248	3.1	-28	48	-10
11	Frontal_Inf_Oper_L	736	3.9	-48	14	4
12	Frontal_Inf_Oper_R	168	3.0	58	10	4
13	Frontal_Inf_Tri_L	2008	4.2	-48	16	4
15	Frontal_Inf_Orb_L	560	3.5	-20	8	-20
16	Frontal_Inf_Orb_R	568	3.5	38	20	-20
17	Rolandic_Oper_L	24	2.5	-48	8	0
18	Rolandic_Oper_R	1368	3.2	52	-2	4
21	Olfactory_L	520	3.6	-20	6	-18
22	Olfactory_R	320	3.4	22	12	-18
**Limbic system**						
29	Insula_L	3288	4.7	-36	-14	-20
30	Insula_R	2024	3.8	38	14	-28
37	Hippocampus_L	88	2.7	-26	-6	-20
39	ParaHippocampal_L	440	4.0	-24	4	-28
41	Amygdala_L	984	3.2	-20	2	-20
**Basal ganglia**						
71	Caudate_L	1072	4.5	-10	10	2
72	Caudate_R	904	4.2	14	12	4
73	Putamen_L	3960	4.7	-26	12	6
74	Putamen_R	3928	5.0	30	-2	2
75	Pallidum_L	144	4.0	-16	6	-4
76	Pallidum_R	496	4.0	26	-2	2
**Temporal lobe**						
81	Temporal_Sup_L	1008	4.3	-46	2	-12
82	Temporal_Sup_R	1272	3.3	54	-2	4
83	Temporal_Pole_Sup_L	2320	4.0	-48	6	-12
84	Temporal_Pole_Sup_R	2720	4.7	42	20	-24
85	Temporal_Mid_L	328	4.1	-50	2	-24
87	Temporal_Pole_Mid_L	32	2.5	-38	8	-28
88	Temporal_Pole_Mid_R	224	3.2	48	16	-26

Table S7: Increased right amygdala connectivity in left bMTLE patients compared to HC

AAL anatomical area		Activated (mm^3)	Z max	MNI Coordinates		
				X	Y	Z
**Limbic system**						
34	Cingulum_Mid_R	32	2.4	8	-50	36
36	Cingulum_Post_R	16	2.4	12	-48	30
**Parietal lobe**						
60	Parietal_Sup_R	1216	3.8	42	-42	56
62	Parietal_Inf_R	440	3.6	40	-40	56
67	Precuneus_L	960	2.9	2	-62	46
68	Precuneus_R	5112	3.8	10	-60	44
**Occipital lobe**						
46	Cuneus_R	104	3.1	20	-64	38
50	Occipital_Sup_R	128	3.3	22	-62	36

Table S8: Decreased right amygdala connectivity in right bMTLE patients compared to HC

AAL anatomical area		Activated	Z max	MNI Coordinates		
				X	Y	Z
**Frontal lobe**						
14	Frontal_Inf_Tri_R	856	3.6	56	26	2
15	Frontal_Inf_Orb_L	160	3.3	-30	14	-22
16	Frontal_Inf_Orb_R	504	3.6	54	24	-4
**Limbic cortex**						
29	Insula_L	832	4.3	-36	-14	-4
30	Insula_R	1808	3.4	44	24	-2
37	Hippocampus_L	696	3.2	-32	-18	-12
38	Hippocampus_R	296	3.4	30	-24	8
39	ParaHippocampal_L	720	3.7	-20	-38	-12
40	ParaHippocampal_R	208	3.6	20	-40	-12
**Basal ganglia**						
73	Putamen_L	712	4.9	-32	-16	-4
74	Putamen_R	1952	3.6	24	6	6
76	Pallidum_R	552	3.5	22	4	2
**Temporal lobe**						
47	Lingual_L	384	3.2	-18	-40	-10
48	Lingual_R	192	3.7	22	-44	-10
55	Fusiform_L	2360	3.5	-20	-32	-22
56	Fusiform_R	928	4.4	24	-46	-12
81	Temporal_Sup_L	1080	4.2	-42	0	-12
82	Temporal_Sup_R	1336	3.4	52	0	-8
83	Temporal_Pole_Sup_L	568	4.0	-40	2	-14
84	Temporal_Pole_Sup_R	176	3.4	52	2	-6
85	Temporal_Mid_L	80	2.7	-46	-14	-14
86	Temporal_Mid_R	144	3.4	68	-16	-8

Table S9: Increased right amygdala connectivity in right bMTLE patients compared to HC

AAL anatomical area		Activated (mm3)	Z max	MNI Coordinates		
				X	Y	Z
**Occipital lobe**						
43	Calcarine_L	480	3.6	2	-68	20
44	Calcarine_R	504	3.4	4	-68	18
45	Cuneus_L	1536	3.6	-8	-76	20
46	Cuneus_R	1384	3.5	4	-68	20
49	Occipital_Sup_L	1008	3.6	-26	-72	22
51	Occipital_Mid_L	1896	3.4	-32	-66	28
**Parietal lobe**						
67	Precuneus_L	16	2.5	0	-64	18
68	Precuneus_R	176	3.3	4	-70	22
